# Diosgenin Alleviates Neuropathic Pain Associated with Alterations in Spinal Mitochondrial and ER Stress-Related Factors in CCI Mice

**DOI:** 10.3390/ijms27198624

**Published:** 2026-09-26

**Authors:** Md. Mahbubur Rahman, Taesu Yoon, Seung Hoon Yum, Ye Won Shim, Youn Yi Jo, Chul-Kyu Park

**Affiliations:** 1 Gachon Pain Center and Department of Physiology, Gachon University College of Medicine, Incheon 21999, Republic of Korea; mahbub84@gachon.ac.kr (M.M.R.); sun991217@gachon.ac.kr (T.Y.); markyum@gachon.ac.kr (S.H.Y.); shimyewon0220@gmail.com (Y.W.S.); 2 Department of Anesthesiology and Pain Medicine, Gachon University, Gil Medical Center, Incheon 21565, Republic of Korea

**Keywords:** diosgenin, neuropathic pain, mitochondrial dysfunction, ER stress, neuroinflammation, apoptosis

## Abstract

Neuropathic pain is difficult to treat as current therapies are often insufficient and provide incomplete relief. However, the mechanisms of chronic nerve injury are poorly addressed. In the present study, we explored the therapeutic effects of natural steroidal saponin diosgenin and its possible spinal mechanisms in a mouse model of chronic constriction injury (CCI)-induced neuropathic pain. C57BL/6 mice underwent CCI and were orally administered diosgenin once daily from three days after surgery. Diosgenin significantly alleviated CCI-induced mechanical allodynia and thermal hyperalgesia and enhanced motor performance in paw withdrawal, open-field, and rotarod tests. These effects were associated with the restoration of mitochondrial homeostasis as evidenced by the increased mitochondrial fusion, decreased fission and activation of NRF2 signaling. Diosgenin also suppressed spinal endoplasmic reticulum (ER) stress, mitochondria apoptotic signaling and the expression of pro-inflammatory cytokines, and pain-related neuropeptides. Mechanistically, the inhibition of spinal NRF2 proteins and the pharmacological induction of ER stress reduced the analgesic effect of diosgenin, highlighting the critical involvement of these pathways in its therapeutic efficacy. Altogether, diosgenin alleviated neuropathic pain in CCI mice that were pharmacologically associated with the restoration of spinal mitochondrial function and ER stress suppression. Therefore, diosgenin could be a promising natural steroidal therapeutic candidate in alleviating neuropathic pain.

## 1. Introduction

Neuropathic pain (NP), arising from lesions or diseases affecting the somatosensory nervous system, represents a formidable clinical challenge affecting an estimated 580 to 830 million people globally, with prevalence rates exceeding 10% in population-based studies [1,2]. Unlike nociceptive pain, which serves a protective function, neuropathic pain is characterized by persistent allodynia, hyperalgesia, and spontaneous pain that significantly impairs quality of life and functional capacity [3,4]. Despite the advances in neuropharmacology, current first-line therapies, including gabapentinoids, serotonin-norepinephrine reuptake inhibitors, and tricyclic antidepressants, provide adequate relief in less than 50% of patients and are frequently associated with dose-limiting side effects such as somnolence, dizziness, respiratory depression, and dependency risk with opioid co-administration [3,5]. This therapeutic gap underscores the urgent need for novel mechanistically distinct agents capable of addressing the multifaceted pathophysiology of chronic neuropathic pain.

Emerging evidence highlights the critical role of neurosteroids, endogenous or synthetic steroids that rapidly modulate neuronal excitability, in pain processing through the allosteric modulation of γ-aminobutyric acid type A (GABAA) receptors, N-methyl-D-aspartate (NMDA) receptors, and T-type calcium channels [6,7]. These steroid-sensitive pathways are integral to central sensitization and descending pain modulation, making neurosteroids promising candidates for neuropathic pain interventions. Unlike synthetic corticosteroids, which induce genomic effects through nuclear receptor activation and carry the risk of metabolic dysregulation and immunosuppression, naturally derived phytosteroids offer rapid membrane-mediated signaling with favorable safety profiles [8].

Diosgenin is a natural steroidal sapogenin found in various plant species, including *Dioscorea* spp., *Smilax* spp., *Trigonella* spp., *Costus* spp., *Aletris* spp., and *Trillium* spp. [9]. Research has shown the diverse pharmacological effects of diosgenin, including immune-modulation, anti-inflammatory properties, antioxidant activity, anti-apoptotic, antidepressant, and anti-nociceptive properties [10,11]. Numerous investigations have revealed that diosgenin possesses preventative and therapeutic properties against a range of neurological diseases [12,13]. Previous in-vivo studies have reported the analgesic effects of diosgenin by up-regulating the Nrf2/HO-1 signaling pathway in diabetic neuropathic pain models [14], reducing oxidative stress, and restoring anti-oxidative activity [15,16] in chronic constriction injury (CCI) models. In our previous study we found that CCI induced neuropathic pain by antagonizing TRPV1 and the regulation of the intracellular Ca^2+^ influx in the dorsal root ganglion [9]. However, the regulatory mechanisms of the effects of diosgenin on neuropathic pain are inadequately documented.

Alterations in spinal signaling play critical roles in the pain regulation and transition to chronic pain [17,18,19] particularly spinal mitochondrial dysfunction [20,21] and endoplasmic reticulum (ER) stress [22,23]. Mitochondrial dysfunction and ER stress are two factors that disrupt cellular homeostasis, resulting in spinal neuroinflammation and neuroapoptosis leading to progressive tissue damage and neurological deficits [24]. Therefore, the objective of this study was to evaluate the effects of diosgenin on spinal signaling alterations, including ER stress and mitochondrial function in a mouse model of CCI induced neuropathic pain and to determine whether these changes are associated with an improvement in pain related behaviors and motor function.

## 2. Results

### 2.1. Effects of Oral Diosgenin Administration on Pain Behaviors in Mice

At baseline (day 0), there were no significant differences in PWT between mouse groups. However, significant decreases in PWT were observed in all groups three days after CCI surgery. Therefore, treatment was initiated on day 3. Our analysis revealed that treatment with diosgenin significantly increased the PWT from day 5 to the end of day 10, whereas no improvement was observed in the vehicle-treated group (Figure 1B). The PWTs in NC, CCI, DG50, and DG100 treated groups on day 10 were 1.36 ± 0.12 g, 0.52 ± 0.07 g, 1.23 ± 0.11 g, and 1.31 ± 0.13 g, respectively. PWT was significantly higher in both diosgenin-treated groups than in the CCI group. No significant differences were observed between these two groups on day 10, suggesting that both 50 mg/kg and 100 mg/kg diosgenin were therapeutically effective in reducing pain sensitivity in the mouse model of CCI-induced pain.

To evaluate the analgesic effect of diosgenin in this model, thermal hyperalgesia was assessed using Hargreaves’ test. On day 10, the PWL values of the NC, CCI, DG50, and DG100 groups were 11.22 ± 0.40 s, 5.82 ± 0.41 s, 9.71 ± 0.34 s, and 11.27 ± 0.38 s, respectively. Diosgenin administration significantly reduced the heat hypersensitivity from days 5 to 10 after injection (Figure 1D) compared to the CCI group. Notably, although both doses were effective, the PWL in the DG 50 mg/kg group remained significantly lower than that in the NC group, whereas the PWL in the 100 mg/kg DG group was comparable to that in the NC group. These findings suggest that 100 mg/kg diosgenin exhibited a greater efficacy in alleviating thermal hyperalgesia.

### 2.2. Effects of Oral Diosgenin Administration on Pain Related Locomotor Activity in Mice

The analgesic effect of diosgenin was further evaluated using open-field locomotor activity by measuring the total traveled distance and traveling velocity. At baseline, no significant differences were observed in the traveled distance and velocity between the mouse groups. However, significant decreases in the traveled distance and traveling velocity were observed in all groups three days after CCI. Our analysis revealed that treatment with diosgenin significantly increased the traveled distance and velocity from day 5 to the end of day 10, whereas no improvement was observed in the CCI group. The traveled distance of the NC, CCI, DG50, and DG100 groups at day 10 were 3174.97 ± 132.15, 2223.48 ± 164.73, 2603.48 ± 227.66, and 3150.30 ± 87.37, respectively. The velocity in these groups was 5.23 ± 0.28, 2.77 ± 0.30, 4.05 ± 0.16, and 5.17 ± 0.24, respectively. The traveled distance and traveling velocity were significantly higher in the diosgenin (50 and 100 mg/kg) groups than in the CCI group. No significant differences were observed between the NC and diosgenin-treated groups on day 10, suggesting that both doses of diosgenin were therapeutically effective in reducing pain sensitivity and improving locomotor activity in the mouse model of CCI-induced pain (Figure 2B,D). Consistent results were also reflected in female CCI mice (Figure S1) indicating sex-independent therapeutic effects.

Pain-related behavior was also evaluated using the rotarod locomotor test. Consistently, the results revealed that treatment with diosgenin significantly increased the latency to fall from day 5 to the end of day 10, while no improvement was observed in the CCI group (Figure 2E,F). The latency to fall of the NC, CCI, DG50, and DG100 at day 10 were 91.36 ± 3.90, 55.02± 3.93, 78.52 ± 3.22, and 84.07 ± 2.48, respectively. The latency to fall was significantly higher in both diosgenin (50 and 100 mg/kg)-treated groups than in the CCI group. No significant differences were observed between the NC, DG50, and DG100 groups on day 10, suggesting that both the DG50 and DG100 groups were therapeutically effective in reducing pain sensitivity in the CCI group. However, the reduction in LF in the DG50 group on day 10 was significantly lower (*p* < 0.052) than that in the NC group, suggesting that DG100 group has more potential in mice for long-term neuropathic pain management.

### 2.3. Effects of Diosgenin on Mitochondrial Function Gene Expression and ER Stress-Related Gene Expression

The expression of mitochondrial dynamics-related genes, including Mfn1, Mfn2, Drp1, and Fis1, was evaluated in spinal cord tissue. In the CCI group, the Mfn1 and Mfn2 expression levels were significantly decreased, whereas the Drp1 and Fis1 expression levels were markedly increased, indicating mitochondrial dysfunction characterized by impaired fusion and enhanced fission. Treatment with diosgenin significantly reversed these alterations in Mfn1, Drp1, and Fis1 suggesting the restoration of mitochondrial homeostasis. A similar trend toward normalization was also observed for XBP1 expression, although this change was less pronounced. Additionally, we assessed the expression of NRF2, a key regulator of oxidative stress. NRF2 expression was significantly decreased in the CCI group and markedly upregulated following diosgenin treatment (Figure 3A). Consistently, a Western blot analysis confirmed that NRF2 protein levels were reduced in the CCI group but significantly increased after diosgenin treatment (Figure 3B). Although total NRF2 protein/mRNA levels serve as an indicator of NRF2 baseline restoration rather than direct proof of nuclear translocation, these findings suggested that diosgenin alleviates neuropathic pain, at least in part, by regulating mitochondrial dysfunction and oxidative stress pathways.

The expression of ER stress-related genes, including GRP78, CHOP, and XBP1, was also assessed in spinal cord tissue. Compared with the NC group, the CCI group exhibited significantly increased expression of these markers, indicating the activation of ER stress pathways. Notably, treatment with diosgenin significantly suppressed the CCI-induced upregulation of the transcriptional level of the GRP78 and CHOP expression. A similar trend toward a reduced expression was also observed for XBP1, although this change was less pronounced (Figure 4A). Protein level changes in representative markers GRP78 confirmed that the protein expression was markedly elevated in the CCI group, whereas diosgenin treatment significantly reversed this increase, further supporting its role in suppressing ER stress (Figure 4B).

### 2.4. Effects of Diosgenin on Apoptosis-Related Gene Expression

To evaluate the antiapoptotic effects of diosgenin, the expression levels of Bax and Bcl-2 in the spinal cord were assessed. In the CCI group, the mRNA expression of the pro-apoptotic gene Bax was significantly increased, whereas that of the anti-apoptotic gene Bcl-2 was significantly decreased compared to the NC group. Diosgenin treatment significantly reversed these effects (Figure 5A). These findings suggested that diosgenin suppressed neuronal apoptosis in the spinal cord. To investigate the involvement of the mitochondrial apoptotic pathway, the transcriptional level of Cytochrome C (Cyt C), Apaf-1, and caspase-9 expression was evaluated. Cyt C, Apaf-1, and caspase-9 levels significantly increased in the CCI group, indicating the activation of mitochondria-mediated apoptosis in CCI-induced neuropathic pain. These changes were significantly attenuated in the DG100 group. Consistently, protein level changes in representative markers Cyt C confirmed that the protein expression was markedly elevated in the CCI group, whereas diosgenin treatment significantly reversed this increase, further supporting its anti-apoptotic effects (Figure 5B).

### 2.5. Effects of Diosgenin on Inflammation and Pain-Related Neuropeptides Gene Expression in Spinal Cord Tissues

The expression of pro-inflammatory cytokines, including tumor necrosis factor-alpha (TNF-α), interleukin (IL)-1β, and IL-6, was significantly increased in the CCI group compared with the NC group, and this elevation was markedly attenuated by diosgenin treatment (Figure 6). The expression levels of pain-related neuropeptides, including CGRP and NGF, were also evaluated. Compared with the NC group, the CCI group exhibited significantly elevated mRNA expression of both markers. Diosgenin treatment (100 mg/kg) significantly attenuated the CCI-induced upregulation of the NGF expression, while the CGRP expression also showed a decreasing trend, although the reduction was less pronounced. (Figure 6). These findings suggested that diosgenin alleviates neuropathic pain, at least in part, by suppressing neuroinflammation and reducing the expression of pain-related neuropeptides in the spinal cord.

### 2.6. Spinal ER Stress and Mitochondrial NRF2 Blockade Reverses Diosgenin-Induced Analgesia

The intrathecal injection of diosgenin 30 μg (Figure S3) produced robust and time-dependent analgesia, whereas, in the co-treatment with thapsigargin (1 µg), ML385 (0.5 µg) partly abolished the analgesic effect of diosgenin. However, the co-treatment with the combination thapsigargin and ML385 totally abrogated the anti-nociceptive response (Figure 7). These results suggest that the alleviation of neuropathic pain by diosgenin is mediated by the suppression of the spinal ER stress and the promotion of mitochondrial function activation. These results also reflect pharmacological inhibition rather than genetic knockout validation.

## 3. Discussion

The present study demonstrated that diosgenin effectively attenuated neuropathic pain-associated behavioral hypersensitivity and improved the pain-associated locomotor dysfunction in a mouse model of CCI. These behavioral improvements were accompanied by the restoration of spinal mitochondrial homeostasis, the suppression of ER stress, the attenuation of apoptotic signaling, and the reduction in spinal neuroinflammation. Specifically, diosgenin treatment reversed the CCI-induced imbalance in spinal mitochondrial fusion/fission dynamics, upregulated the NRF2 antioxidant pathway, and inhibited the inflammatory cascade. Additionally, intrathecal diosgenin delivery produced robust and time-dependent analgesia. However, the cotreatment with the combination of thapsigargin and ML385 completely abrogated the anti-nociceptive response of diosgenin. This finding suggests that the inhibition of spinal ER stress and the activation of mitochondrial function are the mechanisms by which diosgenin attenuates neuropathic pain. Collectively, these findings indicate that diosgenin exerts analgesic effects against neuropathic pain by modulating spinal mitochondrial dysfunction and ER-stress -associated pathways.

To understand the pharmacological significance of these findings, it was essential to consider the structural and biochemical characteristics of diosgenin. This structural similarity to endogenous steroids, including progesterone, corticosteroids, and sex hormones [8,25], allows diosgenin to interact with cellular membranes and nuclear receptors, potentially mimicking or modulating the neuro-steroid signaling pathways involved in nociception. Neuro-steroids are synthesized de novo in the nervous system and modulate pain transmission by interacting with GABAA receptors, NMDA receptors, and other ion channels [6,7]. Compared with synthetic corticosteroids, diosgenin offers superior safety and oral bioavailability, supporting its potential for the long-term management of neuropathic diseases [12]. In experimental studies, diosgenin administered orally easily crosses the blood–brain barrier and reaches the central nervous system, where it demonstrates prolonged retention [26], indicating a long-lasting bioavailability that could explain its prolonged modulation of molecular and cellular pathways in the spinal cord [27,28] and brain [29,30] tissues in neuroinflammatory and injury models.

The strong correlation between pain relief and motor function recovery is a novel and clinically relevant aspect of our behavioral analysis. Patients with neuropathic pain frequently suffer from Kinesiophobia and motor impairments due to guarding behaviors and altered gait mechanics [31]. The alleviation of pain-induced motor suppression is a critical clinical endpoint, as improved mobility is directly linked to a better quality of life and reduced comorbidities in populations with chronic pain [31,32]. In our study, animals treated with diosgenin showed marked improvements in open-field locomotor activity and rotarod performance, which are strongly correlated with a reduced hypersensitivity.

Mitochondrial dysfunction is increasingly being recognized as a central driver of neuropathic pain, contributing to bioenergetic failure and reactive oxygen species (ROS) generation [33]. Our results show that CCI induces a profound disruption in mitochondrial dynamics, characterized by the upregulation of fission markers (Drp1 and Fis1) and downregulation of fusion markers (Mfn1 and Mfn2) [34,35]. The modulation of Drp1 is particularly significant, as the inhibition of mitochondrial fission has been shown to reduce neuropathic pain behavior by limiting oxidative stress [35,36]. This fragmentation impairs the mitochondrial quality and facilitates apoptosis. Diosgenin treatment effectively rebalanced these dynamics, suppressed fission, and promoted fusion. Coupled with the restoration of mitochondrial dynamics, diosgenin exerts powerful antioxidant effects by upregulating the NRF2 pathway. NRF2 is a master transcriptional regulator of antioxidant and mitochondrial defense, and its downregulation is a hallmark of chronic pain [33]. We observed that diosgenin treatment restored NRF2 protein levels in the spinal cord [14]. This mechanism is critical for neutralizing the excessive ROS generated by damaged mitochondria. By enhancing the endogenous antioxidant capacity, diosgenin interrupts the vicious cycle of oxidative stress and mitochondrial damage, thereby protecting spinal neurons from excitotoxicity and sensitization [37]. Our data show a concurrent reduction in pro-inflammatory cytokines IL-1β, IL-6, and TNF-α following treatment. Diosgenin dampens the transcriptional drive for inflammation and prevents the sensitization of nociceptive neurons by glial mediators [38]. Mechanistically, diosgenin appeared to exert its anti-neuroinflammatory effects partially through the NRF2 pathway, as previously suggested [39]. Interestingly, co-treatment with the combination NRF2 inhibitor ML385 with diosgenin suppressed the anti-nociceptive response. These results demonstrate that the alleviation of neuropathic pain by diosgenin might be for the promotion of mitochondrial function activation.

Furthermore, the preservation of mitochondrial integrity by diosgenin directly suppresses the intrinsic apoptotic pathway [40,41]. We found that treatment significantly reduced the release of Cytochrome C and the expression of pro-apoptotic Bax, while restoring anti-apoptotic Bcl-2 levels. Consequently, the activation of caspase-9, the initiator caspase in the mitochondrial apoptotic cascade, is inhibited. The prevention of neuronal apoptosis in the dorsal horn is paramount because the loss of inhibitory interneurons is a key mechanism underlying the inhibition of nociceptive transmission (central sensitization) [40]. By preventing cell death, diosgenin helps maintain an inhibitory tone within the spinal cord, countering the hyperexcitability associated with neuropathic pain.

In addition to mitochondrial protection, our study highlights the critical role of diosgenin in mitigating ER stress. The accumulation of unfolded proteins in the ER lumen triggers the Unfolded Protein Response, a signaling network that, when prolonged, leads to inflammation and apoptosis [42]. We observed elevated levels of the ER stress markers GRP78, CHOP, and spliced XBP1 (sXBP1) in the CCI spinal cord, which were significantly attenuated by diosgenin treatment. GRP78 is a chaperone protein that serves as a sensor for ER stress, whereas CHOP is a pro-apoptotic transcription factor that is induced during severe stress [43]. The reduction in these markers indicates that diosgenin facilitates proper protein folding or enhances the clearance of misfolded proteins, thereby resolving cellular stress. However, the co-treatment with the thapsigargin reduced the anti-nociceptive response of diosgenin. This result demonstrates that the alleviation of neuropathic pain by diosgenin is mediated by the suppression of the spinal ER stress.

Crosstalk between the ER and mitochondria is essential for neuronal homeostasis and apoptotic signaling [24,44]. In persistent pain conditions such as CCI, severe ER stress causes the excessive release of Ca2+ from the ER lumen to the cytosol via IP3R/RyR channels, which is then taken up by the neighboring mitochondria via mitochondria-associated membranes (MAMs) [43]. Mitochondrial Ca2+ overload impairs the electron transport chain, increases the production of reactive oxygen species (ROS), and drives Drp1-mediated mitochondrial fission, eventually resulting in the release of Cytochrome C and the activation of the apoptotic cascade. In contrast, mitochondrial bioenergetic failure causes a shortage of ATP for proper protein folding chaperone activity (e.g., GRP78) in the ER, while, sustained ER stress can exacerbate mitochondrial dysfunction and cellular stress responses, thereby promoting neuronal sensitization and apoptosis thus creating a feed forward cycle of neuroinflammation and central sensitization [44,45]. Therefore, simultaneous regulation of ER stress and mitochondrial dysfunction by diosgenin may contribute to its broad neuroprotective effects under neuropathic conditions [42,43,45]. Importantly, the co-treatment with the combination of thapsigargin and ML385 totally abrogated the anti-nociceptive response. These results suggest that the alleviation of neuro-pathic pain by diosgenin is pharmacologically associated with the concurrent enhancement in spinal mitochondrial function, NRF2 activation, and suppression of ER stress (Figure 8).

The reduction in neuroinflammation was accompanied by the downregulation of pain-related neuropeptides, specifically CGRP and NGF [46,47]. These mediators are closely associated with nociceptive sensitization and persistent pain signaling [47]. Diosgenin exerts neuroprotective effects by regulating NGF and other neurotrophins [48]. Therefore, the ability of diosgenin to lower NGF levels may prevent the maladaptive plasticity that sustains chronic pain [46].

A limitation of the present study is that the critical regulatory events, such as NRF2 nuclear translocation, downstream antioxidant enzyme expression (HO-1/NQO1), XBP1 mRNA splicing, ER stress response (e.g., CHOP, and ATF4) and mitochondrial dynamics markers (Mfn1/2, Drp1) were mainly assessed at the transcript level. Although these qPCR data clearly show transcriptional changes induced by diosgenin, the mRNA expression alone does not confirm functional pathway activation. Crucial stages in these pathways including the IRE1–XBP1 unfolded protein response, caspase-9-mediated apoptotic execution, and mitochondrial fusion/fission are heavily regulated by post-transcriptional splicing, proteolytic processing, and post-translational modifications. Hence, additional studies including nuclear fractionation, protein-level mapping (e.g., spliced XBP1, cleaved caspase-9, and total/phosphorylated Mfn1/2, Drp1, and Fis1) and genetic knockout models (e.g., Nrf2-/- or CHOP-/-) are needed to confirm these mechanisms.

## 4. Materials and Methods

### 4.1. Animals

Six-week-old male C57BL/6 wild-type mice (Orient Bio, Sungnam, Republic of Korea) weighing 18–22 g were used. All experimental animals were housed in a conventional facility under a 12:12-h light/dark cycle (lights on at 8 am) and had ad libitum access to water and chow. Temperature and humidity were maintained at 22 ± 1 °C and 60%, respectively. All animals were acclimated for seven days and allowed to habituate to the new environment for one week prior to any experiment. All behavioral assessments were performed by an experimenter blinded to the treatment groups. All experimental protocols were approved by the Institutional Animal Care and Use Committee of the College of Medicine at Gachon University (Approval No. LCDI-2024-0077).

### 4.2. Chronic Constriction Injury (CCI) Model and Grouping

NP was induced in mice via chronic constriction of the sciatic nerve, as previously described [9]. Briefly, mice were anesthetized with 75 mg/kg pentobarbital sodium (Entobar inj. 100 mg, Hanlim Pharm. Co., Ltd., Seoul, Republic of Korea), the right thigh was incised, blunt cuts were made through the biceps, and the sciatic nerve was exposed. Two silk sutures (7–0; Ailee, Busan, Republic of Korea) were loosely knotted around the full circumference of the sciatic nerve, 2–3 mm apart. This was carefully performed to prevent disruption of the epineural blood supply. The muscle and skin layers were sutured using a silk suture thread (Figure 9A). The normal control group underwent surgery to expose the sciatic nerve; however, the nerve was not ligated to prevent injury.

Three days after surgery, the animals were divided into the following four groups after confirmation of nociception: normal control sham group (NC), vehicle-treated orally; operated CCI group, with orally administered vehicle (3% DMSO); and two diosgenin-treated groups (DG50 and DG100 groups) [9], in which oral diosgenin was administered at doses of 50 and 100 mg/kg. In total, 300 μL of vehicle, diosgenin, were administered orally daily for 9 days (Figure 9B) after assessment of behavioral tests. A total of 40 mice were randomly allocated into two experimental batches within each group (*n* = 5 per batch). One batch was used for assessment of vonFrey and Hargreaves tests whereas second batch was used for evaluation of motor function and locomotory activity using the rotarod and open field tests. Following final behavioral test assessments, spinal cord tissues were collected from randomly selected animals across the two experimental batches in each group for subsequent molecular analysis.

To determine whether induction of ER stress and mitochondrial NRF2 inhibition abolish the therapeutic efficacy of diosgenin 25 mice (*n* = 5) were used. CCI mice were given a single intrathecal injection of diosgenin (30 µg) on day 3, alone or co-administered with pharmacological ER stress inducer, thapsigargin (1 µg), mitochondrial NRF2 inhibitor, ML385 (0.5 µg) or both. Behavioral testing before CCI surgery and after three days before intrathecal injection (0 h) and then 1, 2, 4, 6 and 12 h after injection.

### 4.3. Von Frey and Hargreaves Test

Mechanical allodynia (Figure 1A) [9,49] and thermal hyperalgesia [18,50] (Figure 1C) were measured as previously described at 0, 3, 5, 7, 9, and 10 days before drug administration. These two tests were performed at an interval of 2 h. Mechanical allodynia was measured using von Frey filaments (NC12775-99; North Coast Medical, Morgan Hill, CA, USA). The 50% paw withdrawal threshold (PWT) was calculated using an up-down method. Thermal hyperalgesia was measured by recording paw withdrawal latency (PWL) using a Hargreaves radiant heat apparatus (IITC Life Sciences, Woodland Hills, CA, USA). The cut-off value was set at 20 s to prevent tissue damage.

### 4.4. Open Field Test

Locomotor activity was measured in all experimental mice by evaluating the velocity and distance traveled using an OFT apparatus (Scitech Korea Inc., Seoul, Republic of Korea) [51]. An open field, comprising a square arena (30 × 30 × 30 cm^3^) with four walls and a floor, was placed inside an isolation chamber under low-light illumination. Each mouse was placed in the center of the test field and allowed to explore for 10-min period; and each exploratory session was recorded using a video tracking-based fixed camera placed over the chamber (Ethovision, Noldus Systems, Leesburg, VA, USA) (Figure 2A). Each mouse was habituated to the open field-testing environment and subjected to two explorations (10 min/day) before initiating the experiment at 0, 3, 5, 7, 9, and 10 days before drug administration and locomotor activity at selected time points.

### 4.5. Rotarod Test

The rotarod test was divided into the training and testing stages. On the first training day, the mice were placed on a rotarod (ENV-575M, MED Associates, Fairfax, VT, USA) for 2 min with low-speed rotation (4 rpm). The latency to fall (LF) was recorded in seconds (Figure 2E). Mice that did not remain on the rotarod for two minutes were excluded from the study. On the second training day, the speed of the rotarod system was set to start at 4 rpm and reached a maximum of 40 rpm. The mice were subjected to five rounds of training daily for four consecutive days. Mice that could not remain on the rotor for 250 s were excluded. On the testing days, mice were placed on the rotarod before initiating the drug administration at 0, and following 3, 5, 7, 9, and 10 days just before drug administration. Locomotor activity was measured by recording LF at selected time points.

### 4.6. Quantitative Real-Time Polymerase Chain Reaction (qRT-PCR)

Total RNA was isolated from each group of ipsilateral spinal cord tissue (L3-L6) using TRIZOL Reagent (Ambion, Austin, TX, USA) according to the manufacturer’s protocol. The yield and purity of isolated RNA were determined using a NanoDrop 2000 spectrophotometer (Thermo Scientific, Wilmington, DE, USA). First-strand cDNA was synthesized from 1 μg of total RNA using M-MLV reverse transcriptase kits (Invitrogen, Carlsbad, CA, USA) in a total volume of 20 μL according to the manufacturer’s instructions. RT-qPCR was performed on a QuantStudio 1 (Thermo Fisher Scientific, Waltham, MA, USA) with a 96 well plate using SensiFAST^®^ SYBR Lo-ROX kit (Meridian Bioscience, Cincinnati, OH, USA) and a reaction volume of 20 μL with 1 μL of sample. The following amplification program was used for all qPCRs: 95 °C for 2 min, and 40 cycles of 5 s at 95 °C and 30 s at 60 °C. 2^−ΔΔCt^ values were ana-lyzed with GAPDH as a reference gene. The primers used in this study are listed in Table 1.

### 4.7. Western Blot Analysis

For western blot analysis, the ipsilateral spinal cord tissues (L3–L6) were homogenized in an ice-cold tissue protein extraction reagent (NP-40; EBA-1049; Elpis Biotech, Inc., Daejeon, Republic of Korea). Protein concentrations were determined using a bicinchoninic acid assay kit (23227; Thermo Fisher Scientific). Equal amounts of proteins were separated by sodium dodecyl sulfate–polyacrylamide gel electrophoresis (SDS-PAGE) and electro-transferred onto polyvinylidene fluoride (PVDF) membranes (IPVH00010; Millipore, Burlington, MA, USA). The membranes were blocked with 5% nonfat dry milk in TBST and incubated with the primary antibodies listed in Table 2. After washing with TBST, the membranes were incubated for 1 h at room temperature with the following horseradish peroxidase (HRP)-conjugated secondary antibodies: mouse anti-rabbit IgG-HRP (1:5000; Santa Cruz Biotechnology, sc-2357, Dallas, TX, USA) and mouse IgG HRP-conjugated antibody (1:5000; Bio-Techne, Minneapolis, MN, USA). After washing with TBST, protein bands were visualized using an enhanced chemiluminescence detection system (Beyotime Biotechnology, Shanghai, China). Densitometric analysis was performed using a UVP BioSpectrum imaging system and quantified using Image Quant LAS 4000 software (GE Healthcare Life Sciences, San Diego, CA, USA).

### 4.8. Statistical Analysis

Data are expressed as mean ± SEM. Behavioral data were analyzed using two-way repeated-measures analysis of variance (ANOVA), followed by Bonferroni’s post hoc test. Molecular data were analyzed using a one-way ANOVA followed by Tukey’s post-hoc test. Spearman’s rank correlation coefficients were used to measure correlations [52]. Statistical significance was set at *p* < 0.05.

## 5. Conclusions

In conclusion, the present study demonstrates that diosgenin effectively attenuates peripheral neuropathic pain and improves pain-associated locomotor dysfunction in a mouse model of chronic constriction injury. Spinal ER stress inducer and mitochondrial NRF2 blockade reverses diosgenin-induced analgesia. Collectively, these findings identify the regulation of spinal mitochondrial homeostasis and ER stress as central mechanisms underlying the neuroprotective and analgesic actions of diosgenin, supporting its potential as a promising natural steroidal therapeutic candidate for the long-term management of peripheral neuropathic pain (Figure 8). Although intrathecal inhibitor studies provide functional pharmacological evidence supporting the involvement of NRF2 signaling and ER stress suppression, the data are correlative. Future studies using genetic knockout mouse models (e.g., Nrf2-/- or Chop-/-) will be important for establishing definitive molecular causality.

## Figures and Tables

**Figure 1:**
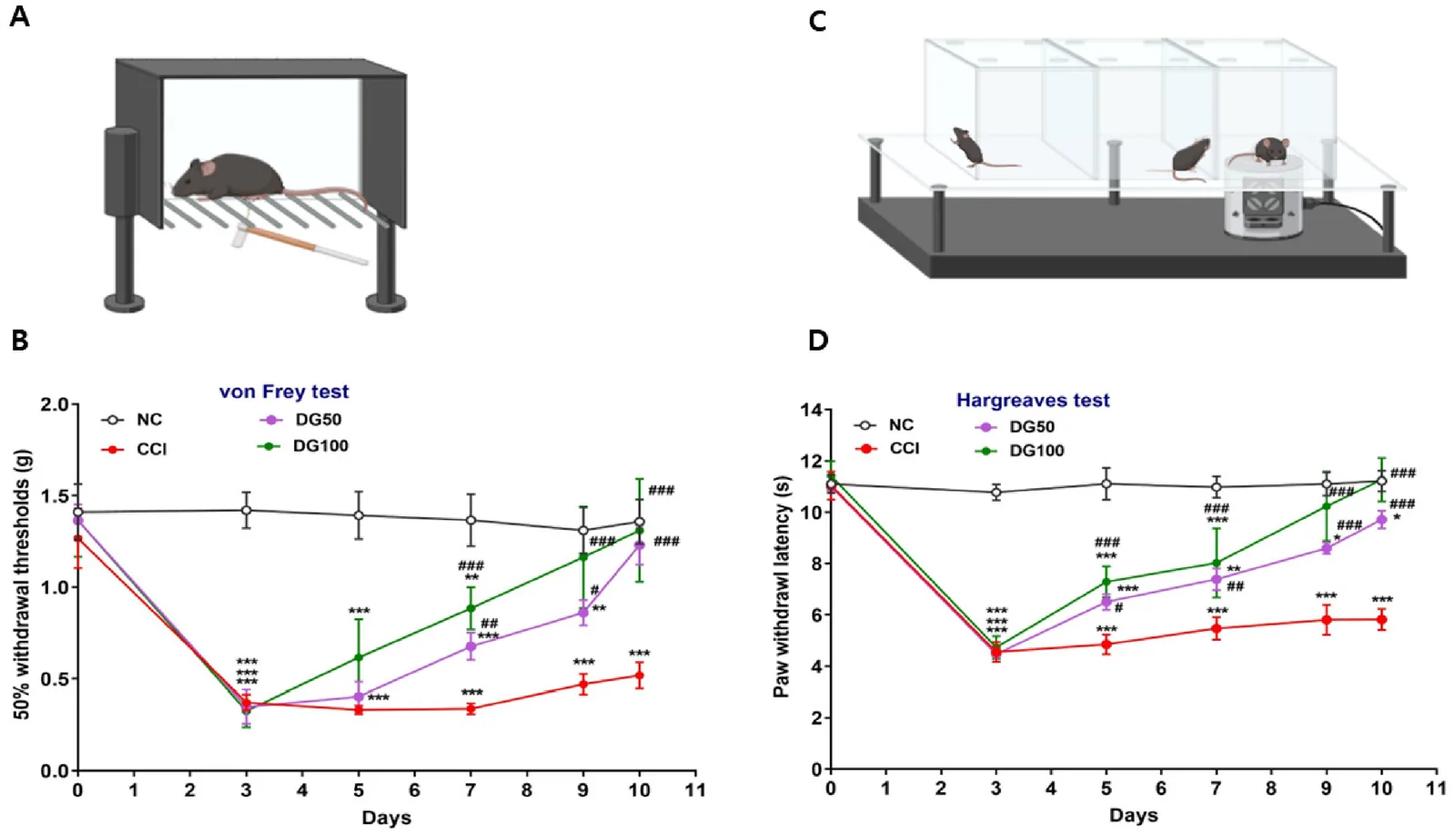
Diosgenin alleviates pain behaviors in a mouse model of chronic constriction injury (CCI). (**A**,**B**) Representative Effects on mechanical allodynia and (**C**,**D**) thermal hyperalgesia are shown. Data are reported as means ± standard error of the mean (*n* = 5). * *p* < 0.05, ** *p* < 0.01, *** *p* < 0.001, repeated-measure multiple comparisons tests by two-way analysis of variance versus the NC group and # *p* < 0.05, ## *p* < 0.01, ### *p* < 0.001 vs. the CCI group.

**Figure 2:**
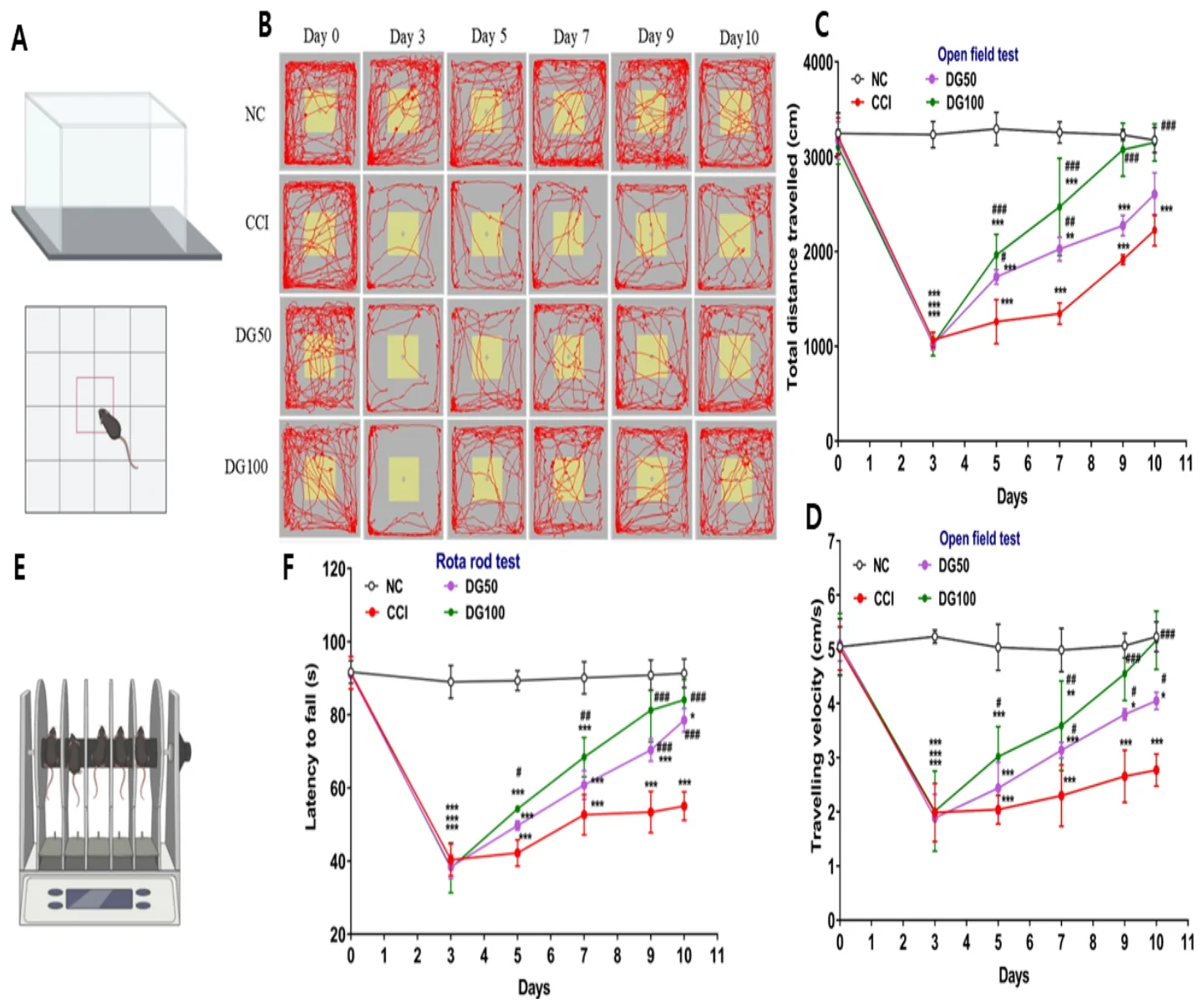
Diosgenin treatment improves locomotor activity in mice model of chronic constriction injury. (**A**) open field; (**B**) representative traces in OFT during a period of 10 min. (**C**) the total distance of mice in the OFT; (**D**) average velocity of mice in the OFT; (**E**) Rotarod, and (**F**), latency to fall in rotarod. Data are reported as means ± standard error of the mean (*n* = 5). * *p* < 0.05, ** *p* < 0.01, *** *p* < 0.001, repeated-measure multiple comparisons tests by two-way analysis of variance versus the NC group # *p* < 0.05, ## *p* < 0.01, ### *p* < 0.001 vs. the CCI group.

**Figure 3:**
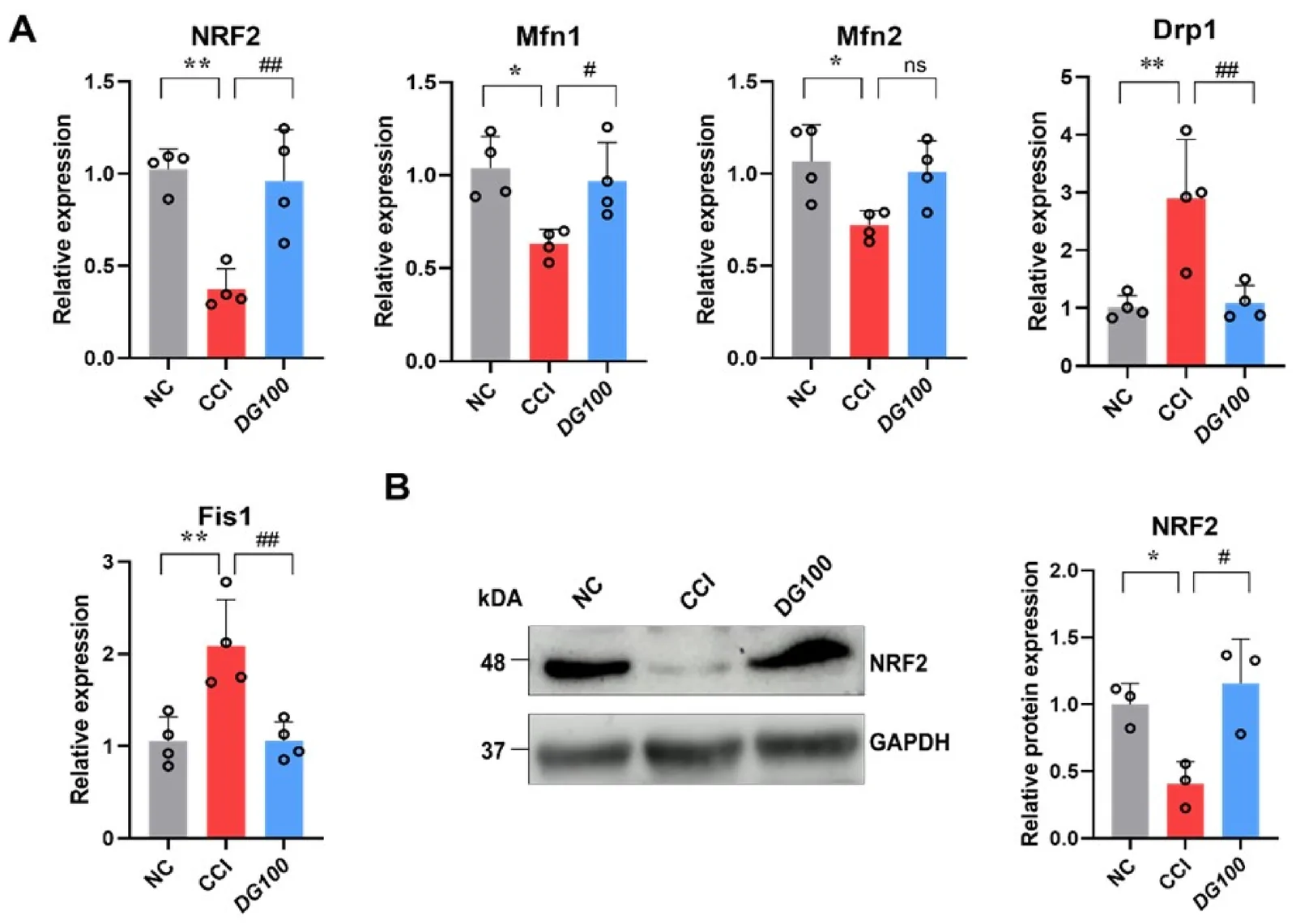
Effects of diosgenin on CCI-induced mitochondrial function in the spinal cord of mice. (**A**) mRNA expression levels of mitochondrial dynamics-related genes (Mfn1, Mfn2, Drp1, and Fis1) and NRF2 in the spinal cord, as determined by qPCR analysis. Data are presented as mean ± SEM (*n* = 4 per group). (**B**) Representative Western blot images and densitometric analysis of NRF2 protein expression in the spinal cord. Band intensities were normalized to GAPDH and expressed as mean ± SEM (*n* = 3 per group). NRF2 expression was significantly decreased in the CCI group and restored by diosgenin treatment. * *p* < 0.05, ** *p* < 0.01 vs. the NC group # *p* < 0.05, ## *p* < 0.01, vs. the CCI group (one-way ANOVA followed by Tukey’s post hoc test).

**Figure 4:**
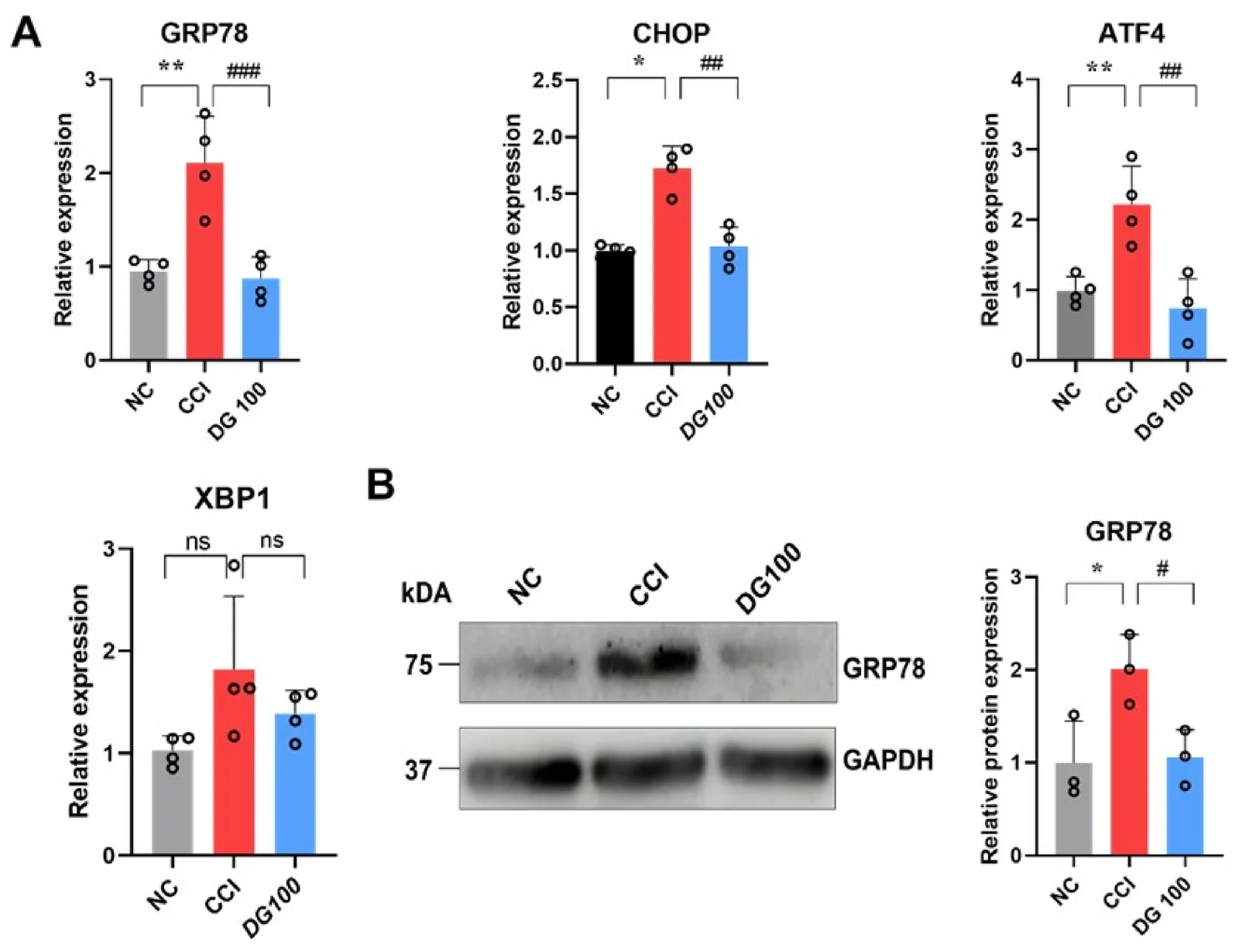
Effects of diosgenin on CCI-induced ER stress in the spinal cord of mice. (**A**) mRNA expression levels of ER stress-related genes (GRP78, CHOP, ATF4, and XBP1) in the spinal cord, as determined by qPCR analysis. Data are presented as mean ± SEM (*n* = 4 per group). (**B**) Representative western blot images and densitometric analysis of GRP78 protein expression in the spinal cord. Band intensities were normalized to GAPDH and expressed as mean ± SEM (*n* = 3 per group). GRP78 expression was significantly increased in the CCI group and markedly reduced by diosgenin treatment. * *p* < 0.05, ** *p* < 0.01 vs. the NC group; # *p* < 0.05, ## *p* < 0.01, ### *p* < 0.001 vs. the CCI group (one-way ANOVA followed by Tukey’s post hoc test).

**Figure 5:**
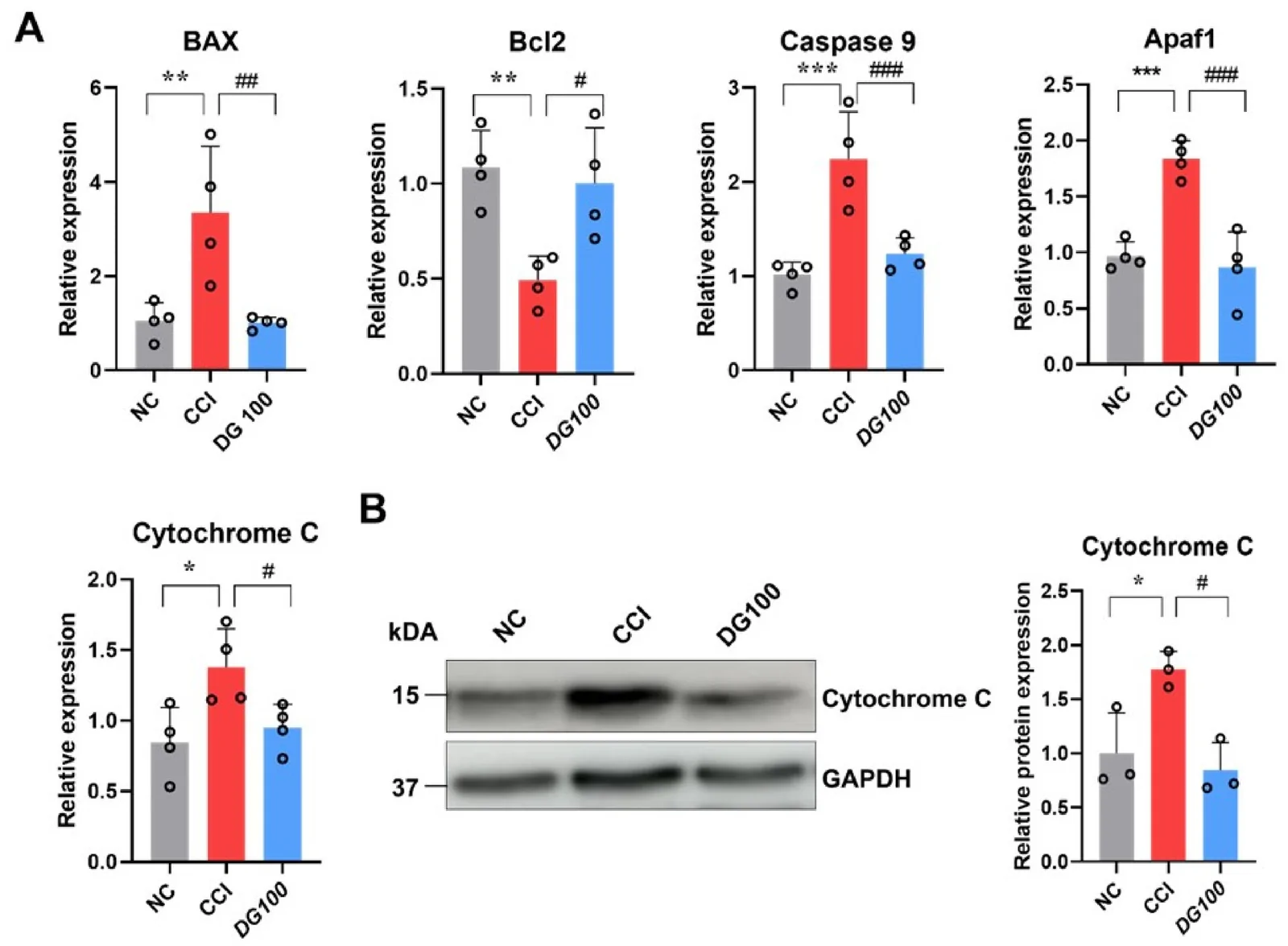
Effects of diosgenin on CCI-induced apoptosis in the spinal cord of mice. (**A**) mRNA expression levels of apoptosis-related genes (Bax, Bcl-2, caspase-9, Apaf-1, and Cytochrome C) in the spinal cord, as determined by qPCR analysis. (**B**) Representative Western blot images and densitometric analysis of cytochrome C (CytC) protein expression in the spinal cord. Band intensities were normalized to GAPDH and expressed as mean ± SEM (*n* = 4 per group). Cytochrome c expression was significantly increased in the CCI group and markedly reduced by diosgenin treatment. Data are presented as mean ± SEM (*n* = 3 per group). * *p* < 0.05, ** *p* < 0.01, *** *p* < 0.001 vs. the NC group; # *p* < 0.05, ## *p* < 0.01, ### *p* < 0.001 vs. the CCI group (one-way ANOVA followed by Tukey’s post hoc test).

**Figure 6:**
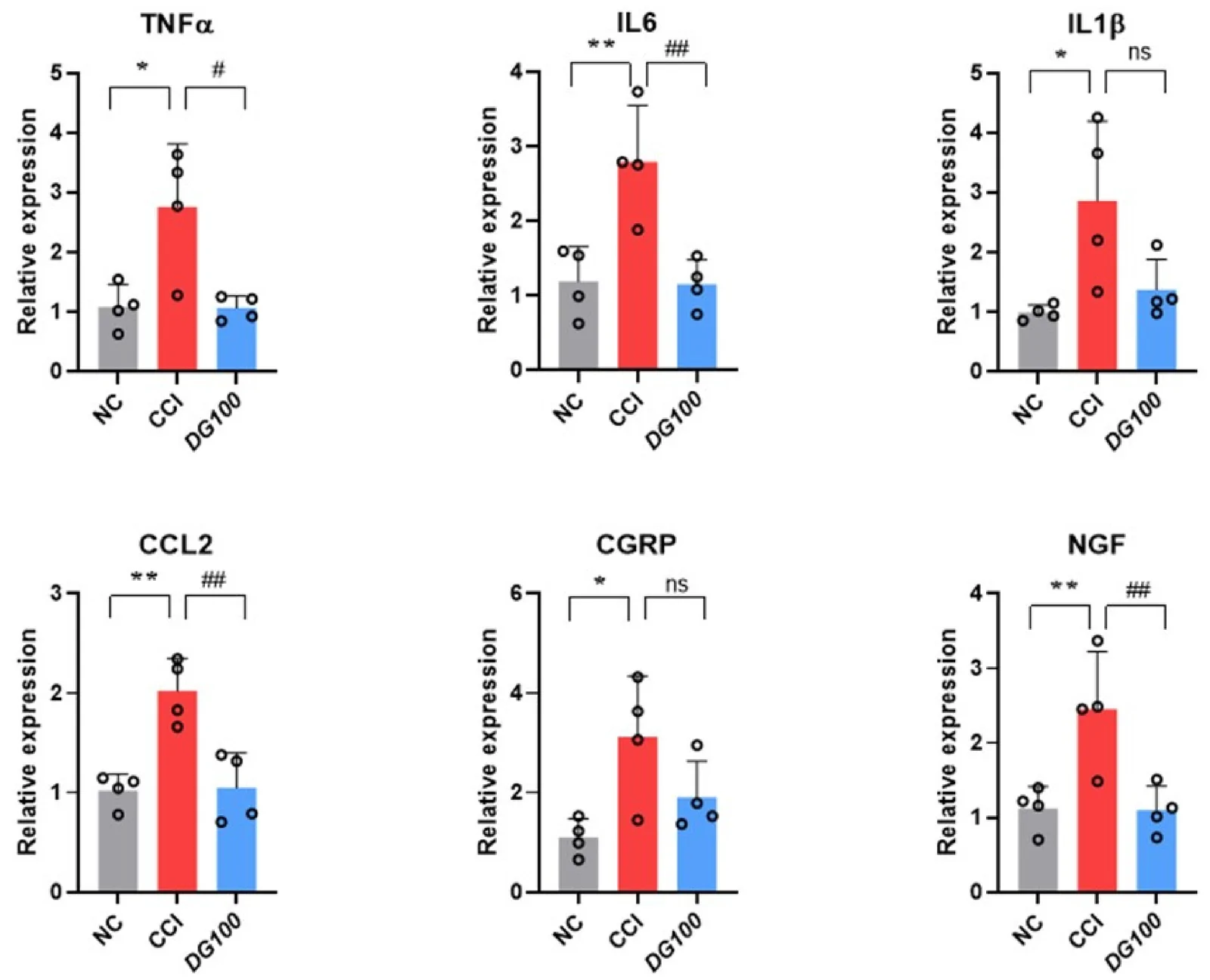
Effects of diosgenin on CCI-induced inflammation in the spinal cord of mice. mRNA expression levels of pro-inflammatory cytokines (TNF-α, IL-6, and IL-1β), chemokine (CCL2), and pain-related neuropeptides (CGRP and NGF) in the spinal cord, as determined by qPCR analysis. Data are presented as mean ± SEM (*n* = 4 per group). * *p* < 0.05, ** *p* < 0.01 vs. the NC group; # *p* < 0.05, ## *p* < 0.01 vs. the CCI group (one-way ANOVA followed by Tukey’s post hoc test). ns, not significant.

**Figure 7:**
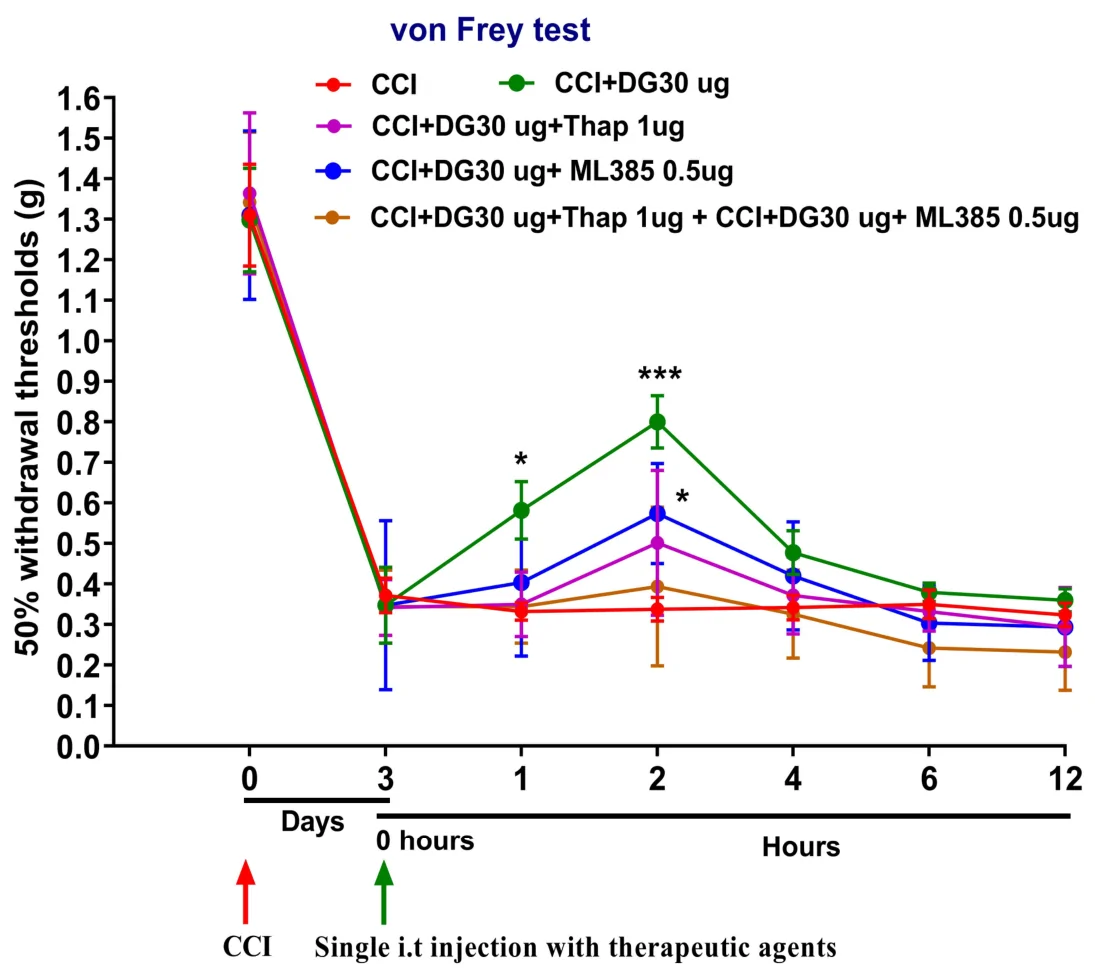
Spinal ER stress inducer (thapsigargin) and NRF2 inhibition abolish the analgesic effect of diosgenin in CCI mice. * *p* < 0.05, *** *p* < 0.001, repeated-measure multiple comparisons tests by two-way analysis of variance versus the NC group. CCI, chronic constriction injury; DG, diosgenin; Thap, thapsigargin.

**Figure 8:**
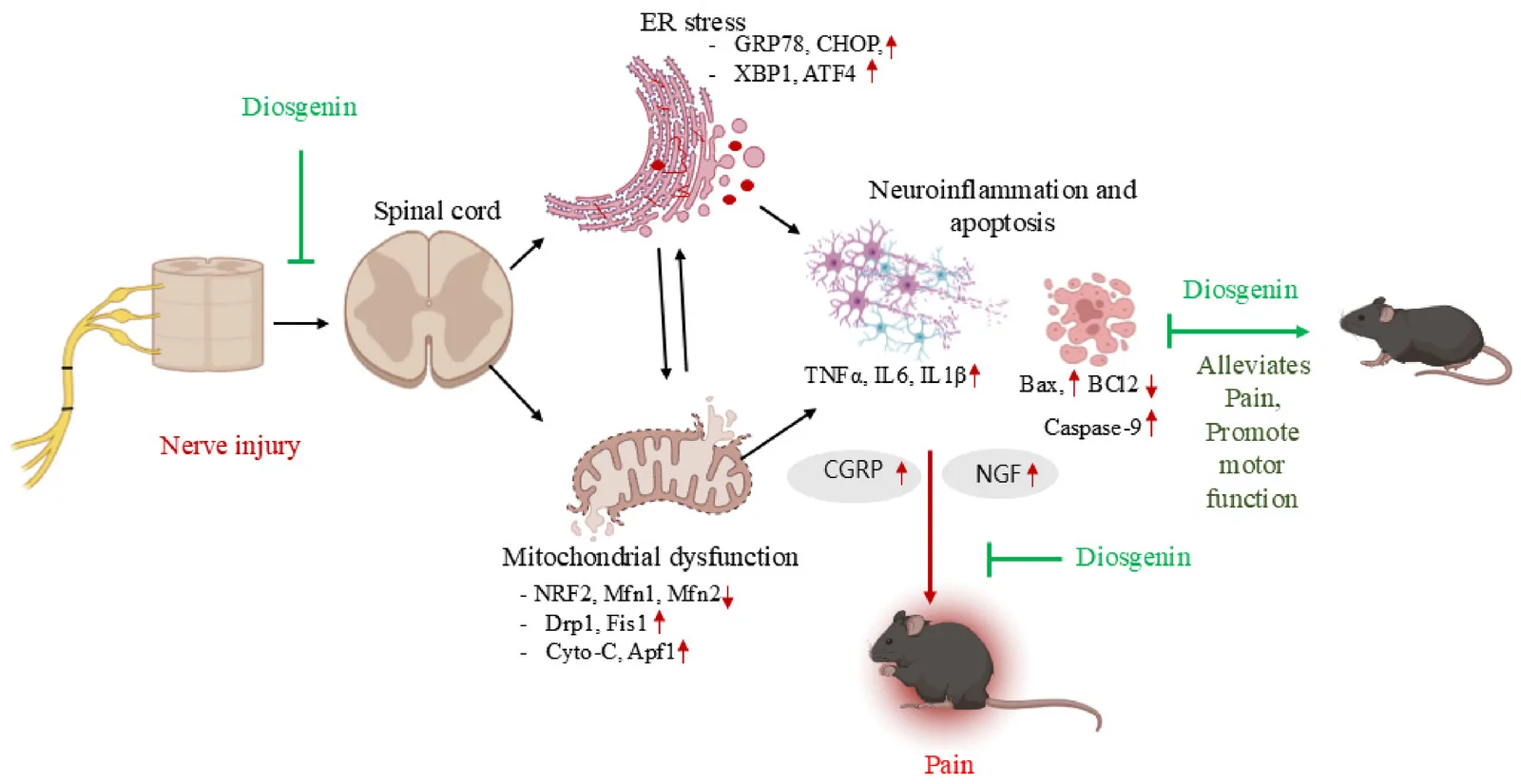
Schematic diagram of the proposed mechanisms by which diosgenin alleviates pain and promotes motor function by reduction in spinal ER stress and preserving mitochondrial function. Administration of diosgenin suppressed ER stress and corrected mitochondrial dysfunction, responsible for reducing neuroinflammation and apoptosis and reduced pain associated neuropeptides, thereby alleviating pain and promoting motor function. Upward arrows (↑) indicate increased expression or signal levels, whereas downward arrows (↓) indicate decreased expression or signal levels.

**Figure 9:**
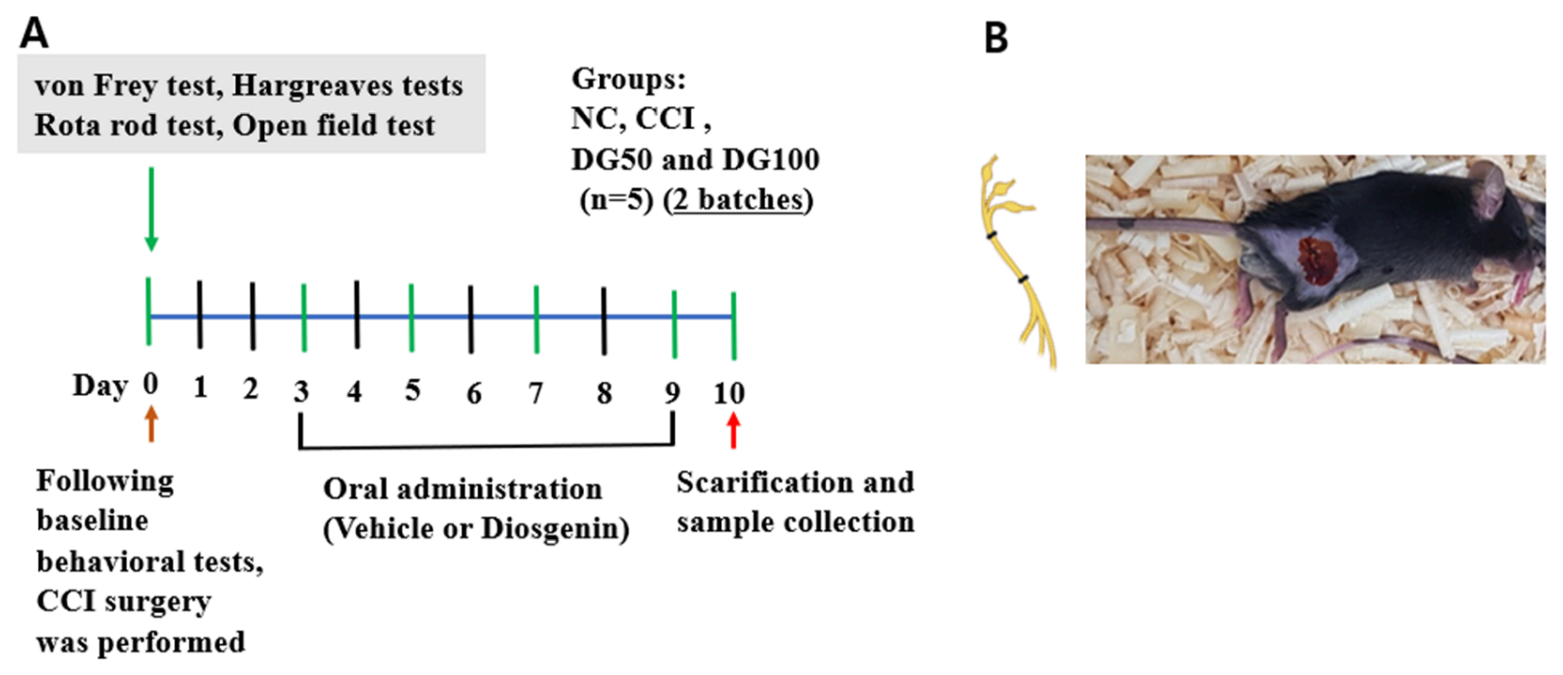
Experimental timeline and methods of chronic constriction nerve injury model in mice. (**A**) Representative image of chronic constriction injury (CCI) of the sciatic nerve in mice, and (**B**) schematic timeline of the experimental procedure, including CCI surgery, behavioral assessments (von Frey and Hargreaves tests), oral administration of diosgenin (50 and 100 mg/kg), and tissue collection.

**Table 1:** Sequences used in the study.

Primer	Sequence (5′-3′)	Tm (℃)	Nucleotide RefSeq	Product Size (bp)
NRF2	F: CAGCATAGAGCAGGACATGGAG R: GAACAGCGGTAGTATCAGCCAG	61	NM_010902.5	107
Mfn1	F: CCAGGTACAGATGTCACCACAG R: TTGGAGAGCCGCTCATTCACCT	62	NM_024200.5	149
Mfn2	F: GTGGAATACGCCAGTGAGAAGC R: CAACTTGCTGGCACAGATGAGC	62	XM_006538609.5	115
Fis1	F: GCTGGTTCTGTGTCCAAGAGCA R: GACATAGTCCCGCTGTTCCTCT	62	NM_025562.3	144
Drp1	F: GCGAACCTTAGAATCTGTGGACC R: CAGGCACAAATAAAGCAGGACGG	62	NM_001276341.2	104
GRP78	F: TGTCTTCTCAGCATCAAGCAAGG R: CCAACACTTCCTGGACAGGCTT	62	NM_022310.3	144
CHOP	F: CACATCCCAAAGCCCTCG R: CGTTCTCCTGCTCCTTCTC	58	NM_007837.5	158
ATF4	F: AACCTCATGGGTTCTCCAGCGA R: CTCCAACATCCAATCTGTCCCG	62	NM_001287180.1	125
XBP1	F: TGGACTCTGACACTGTTGCCTC R: TAGACCTCTGGGAGTTCCTCCA	64	NM_001271730.1	136
Cytochrome C	F: GAGGCAAGCATAAGACTGGACC R: ACTCCATCAGGGTATCCTCTCC	61	NM_007808.5	132
Apaf1	F: CACGAGTTCGTGGCATATAGGC R: GGAAATGGCTGTCGTCCAAGGA	62	NM_001042558.2	113
BAX	F: AGGATGCGTCCACCAAGAAGCT R: TCCGTGTCCACGTCAGCAATCA	64	NM_007527.4	103
Bcl2	F: CCTGTGGATGACTGAGTACCTG R: AGCCAGGAGAAATCAAACAGAGG	60	NM_009741.5	123
Caspase 9	F: GCTGTGTCAAGTTTGCCTACCC R: CCAGAATGCCATCCAAGGTCTC	61	NM_001355176.2	124
CGRP	F: GCACTGGTGCAGGACTATATGC R: CTCAGATTCCCACACCGCTTAG	61	NM_001305616.1	116
TNF- α	F: GGTGCCTATGTCTCAGCCTCTT R: GCCATAGAACTGATGAGAGGGAG	61	NM_001278601.1	139
IL6	F: TACCACTTCACAAGTCGGAGGC R: CTGCAAGTGCATCATCGTTGTTC	62	NM_031168.2	116
CCL2	F: GCTACAAGAGGATCACCAGCAG R: GTCTGGACCCATTCCTTCTTGG	61	NM_011333.3	106
IL-1β	F: TGACGGACCCCAAAAGATGA R: TCTCCACAGCCACAATGAGT	60	NM_008361.4	140
NGF	F: GTT TTG CCA AGG ACG CAG CTT TC R: GTT CTG CCT GTA CGC CGA TCA A	63	NM_001112698.2	111
GAPDH	F: ATGACTCCACTCACGGCAAA R: TGATGTTAGTGGGGTCTCGC	60	NM_001411840.1	111

**Table 2:** Antibodies used in this study.

Antibody	Company (Cat. No.)	Working Concentration
Anti-NRF2	Novus Biologicals (NBP1-32822)	1/1000
Anti-GRP78	Abcam (ab21685)	1/1000
Anti-Cytochrome C	Santa Cruz Biotechnology (sc-13156)	1/500
Anti-GAPDH	Santa Cruz Biotechnology (sc-166545)	1/1000

## Data Availability

Data will be made available on request.

## Supplementary Materials

The following supporting information can be downloaded at: https://www.mdpi.com/article/10.3390/ijms27198624/s1.

## Abbreviations

The following abbreviations are used in this manuscript:

CCI	Chronic Constriction Injury
NRF2	Nuclear Factor Erythroid 2-Related Factor 2
ER	Endoplasmic Reticulum
ROS	Reactive Oxygen Species
NP	Neuropathic Pain
GABAA	Gamma-Aminobutyric Acid Type A Receptor
NMDA	N-Methyl-D-Aspartate Receptor
TRPV1	Transient Receptor Potential Vanilloid 1
NC	Normal Control
DMSO	Dimethyl Sulfoxide
DG	Diosgenin
PWT	Paw Withdrawal Threshold
PWL	Paw Withdrawal Latency
LF	Latency to Fall
OFT	Open Field Test
qRT-PCR	Quantitative Real-Time Polymerase Chain Reaction
RNA	Ribonucleic Acid
cDNA	Complementary Deoxyribonucleic Acid
GAPDH	Glyceraldehyde-3-Phosphate Dehydrogenase
Mfn1	Mitofusin 1
Mfn2	Mitofusin 2
Drp1	Dynamin-Related Protein 1
Fis1	Mitochondrial Fission Protein 1
GRP78	Glucose-Regulated Protein 78
CHOP	C/EBP Homologous Protein
XBP1	X-Box Binding Protein 1
Bax	Bcl-2-Associated X Protein
Bcl-2	B-Cell Lymphoma 2
Cyt C	Cytochrome C
Apaf-1	Apoptotic Protease Activating Factor-1
IL	Interleukin
TNF-α	Tumor Necrosis Factor-Alpha
CGRP	Calcitonin Gene-Related Peptide
NGF	Nerve Growth Factor
DRG	Dorsal Root Ganglion
HO-1	Heme Oxygenase-1
UPR	Unfolded Protein Response
sXBP1	Spliced X-Box Binding Protein 1
ATP	Adenosine Triphosphate
ANOVA	Analysis of Variance
SEM	Standard Error of the Mean
