# Supplementary material for: Diosgenin Alleviates Neuropathic Pain Associated with Alterations in Spinal Mitochondrial and ER Stress-Related Factors in CCI Mice
Source: Int J Mol Sci. 2026 Sep 26;27(19):8624. doi: 10.3390/ijms27198624 (PMC13665389; doi:10.3390/ijms27198624)

Supplementary Figure S1.

Diosgenin alleviates neuropathic behaviors in CCI mice

The mechanisms underlying neuropathic pain are sex-dependent, so it is important to know whether diosgenin works in female neuropathic pain. Additionally, 15 female mice were divided into three groups (n=5); a total of 15 female mice were randomly allocated into three experimental groups (n=5), namely, the NC group, CCI+ vehicle, and CCI+ DG100 (diosgenin-treated group), and were used for the assessment of von Frey and Rotarod tests. Interestingly, diosgenin also attenuated mechanical hyperalgesia and improved pain-associated locomotor activity in female mice (Figure S1) like male mice, suggesting diosgenin alleviates pain in a sex-independent manner.

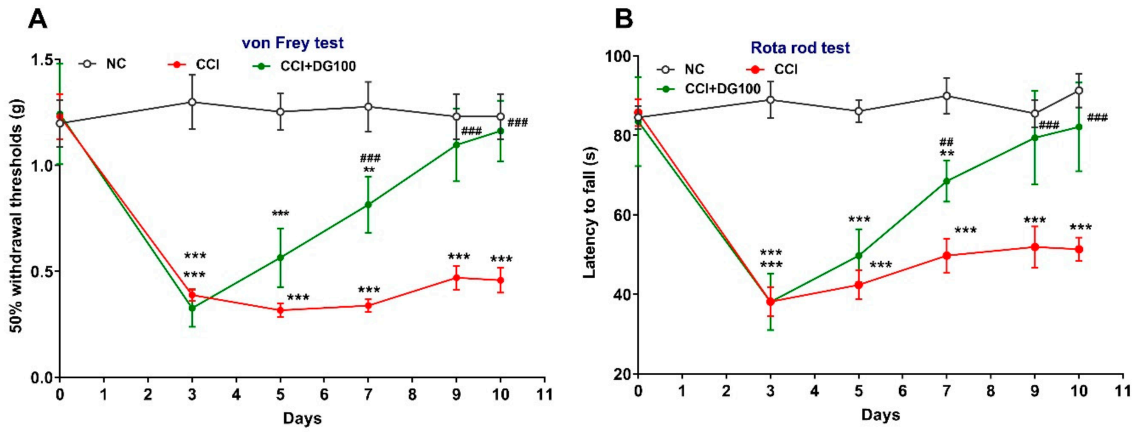

Figure S1. Diosgenin alleviates pain behaviors in a mouse model of chronic constriction injury (CCI) in female mice. (A) Representative effects on mechanical allodynia and (B) latency to fall in rotarod are shown. Data are reported as means ± standard error of the mean (n = 5). *p<0.05, **p<0.01, ***p<0.001, repeated-measure multiple comparisons tests by two-way analysis of variance versus the NC group and ### p<0.001 vs. the CCI group.

Supplemental Figure S2.

Correlation between pain behaviors and locomotor activities markers

The correlation between pain behaviors (PWT and PWL) was evaluated using locomotor activity (total traveled distance, traveling velocity, and latency to fall). The results revealed that the total traveled distance (*r* = +0.8108; *p* < 0.0001), traveling velocity (*r* = +0.7667; *p* < 0.0001), and latency to fall (*r* = + 0.8028; *p* < 0.0001) showed a significant positive correlation with PWT. Consistently, the total traveled distance (r = +0.8308; *p* < 0.0001), traveling velocity (*r* = +0.8165; *p* < 0.0001), and latency to fall (*r* = + 0.8873; *p* < 0.0001) showed a significant positive correlation with PWL (Figure. S2). These findings indicate a strong association between the attenuation of neuropathic pain behaviors and recovery of locomotor activity.

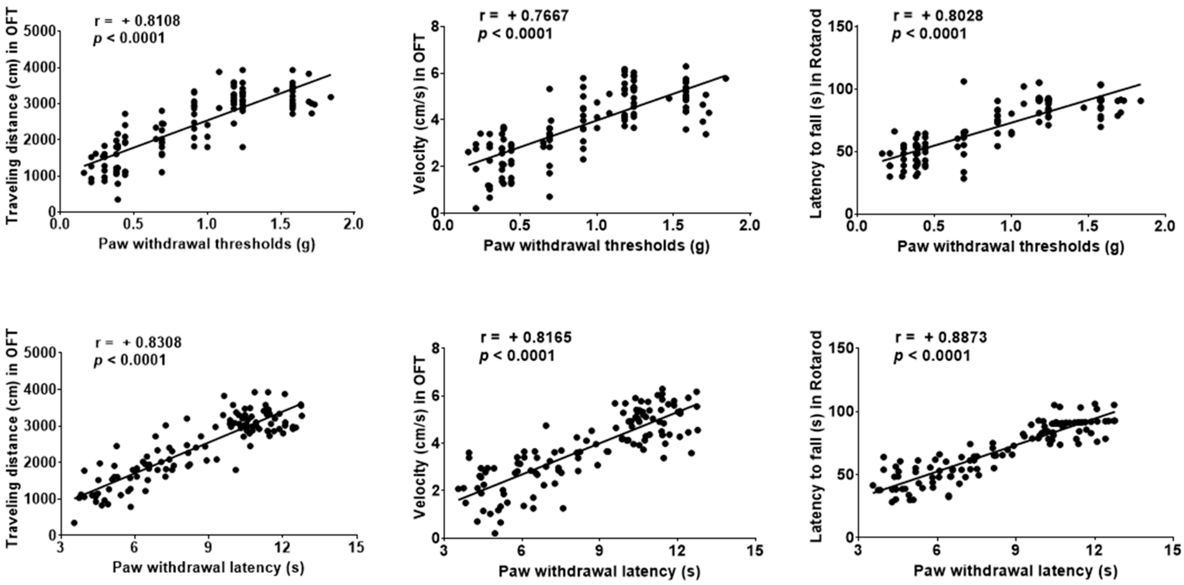

Figure S2. Spearman's rank correlation between pain behaviors (paw withdrawal thresholds and paw withdrawal latency) and locomotor activities (traveling distance, velocity, and latency to fall) in CCI-induced chronic neuropathic pain in mice. n=5 (total 30 variables in each group) and the data of each group and each experimental time points (six) 0, 3, 5, 7, 9, and 10 days were included for Spearman's rank correlation analysis. OFT, open field test.

Supplemental Figure S3

Intrathecal diosgenin dose-dependently alleviates mechanical allodynia in CCI mice

To determine whether a single dose of diosgenin could alleviate CCI-induced pain, diosgenin (20, 30, and 40 ug, i.t.) was given on day three after CCI surgery. The behavioral tests were performed before CCI surgery and after three days before intrathecal diosgenin injection (o hours), and then 1, 2, 4, 6, and 12 hours after diosgenin injection.

We administered three different doses of diosgenin intrathecally in CCI mice to determine the most effective therapeutic dose in mice to alleviate CCI-induced pain. A single dose of diosgenin (20, 30, and 40 ug, i.t.) was given on day three. The behavioral tests were conducted before diosgenin injection (0 h) and 1, 2, 4, 6, and 12 h after injection. The results showed that 30 ug i.t. diosgenin treatment significantly increased the PWTs of CCI mice in a dose-dependent manner and the analgesic effect of diosgenin began at 1 h, reached a peak at 2 h, and lasted for at least 2 h (**Figure S3**). Therefore, 30 ug i.t. is considered the most therapeutically effective dose. Importantly, the 30-ug-i.t.-diosgenin-treated group in normal mice (NC+30ug) did not alter any behavioral changes when compared with the vehicle-treated group (NC group), which suggested that 30 ug i.t. diosgenin is a safe dose.

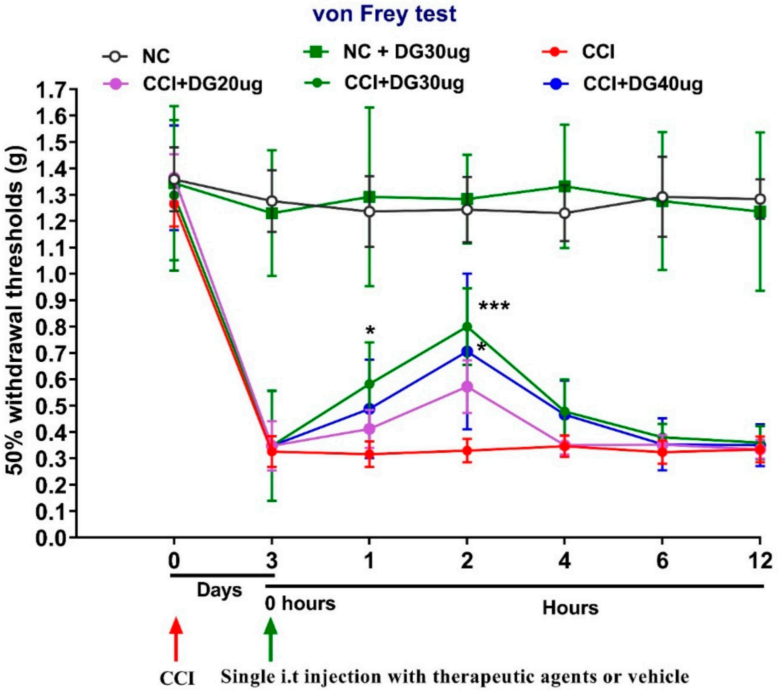

Figure S3. Time-dependent analgesic effects of a single dose of diosgenin (20, 30, and 40 ug, i.t.) on day three after the CCI surgery significantly increased the PWTs of CCI mice. Data are reported as means ± standard error of the mean (n = 5). *p<0.05, **p<0.01, ***p<0.001, repeated-measure multiple comparisons tests by two-way analysis of variance versus the NC group and ### p<0.001 vs. the CCI group.

Supplemental Figure S4.

Uncropped Western blot data images for three repeats

GRP78

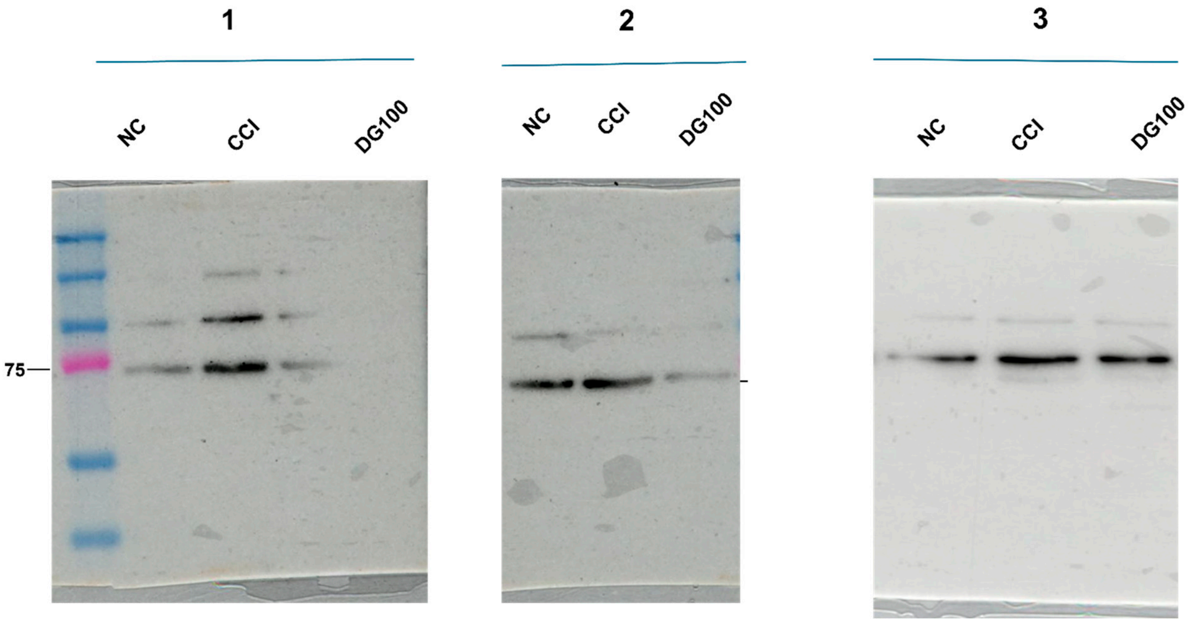

Cytochrome C

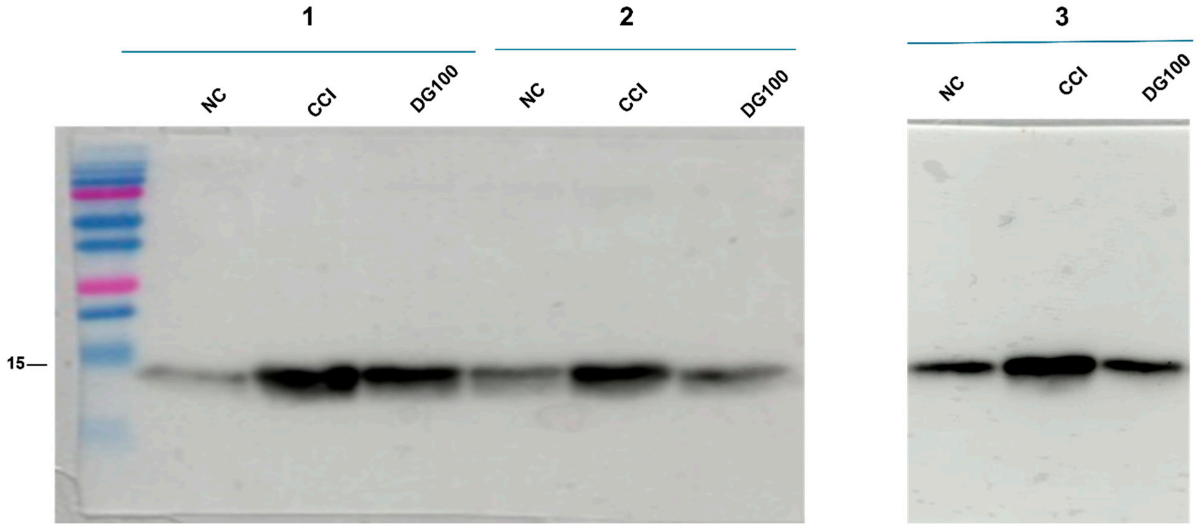

NRF2

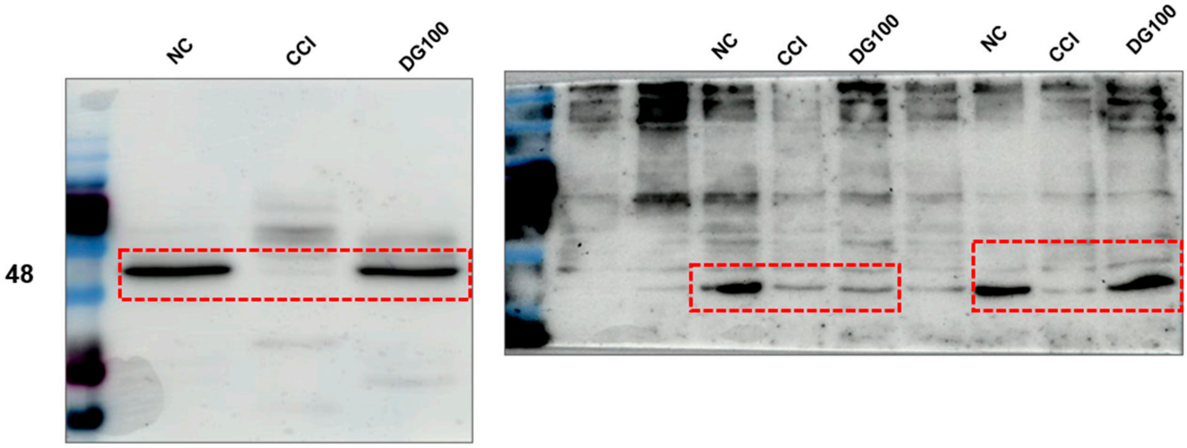

Supplement: Supplementary file 1 [file ijms-27-08624-s001.zip › ijms-4546946-supplementary.pdf]
